# Quality assurance of approved out of programme psychiatry training and research over the past 5 years

**DOI:** 10.1192/pb.bp.114.046722

**Published:** 2015-06

**Authors:** Victoria Osman-Hicks, Hannah Graham, Peter Leadbetter, Andrew Brittlebank

**Affiliations:** 1 Royal College of Psychiatrists Quality Assurance Committee, UK; 2 Southern Health NHS Foundation Trust, Hampshire and Wessex Deanery, UK; 3 Edge Hill University, Liverpool, UK; 4 Northumberland, Tyne and Wear NHS Health Trust.

## Abstract

**Aims and method** This paper intends to analyse the number of applications, trainee demographic and approval rate of those applying for out of programme training (OOPT) or out of programme research (OOPR) between January 2008 and April 2013 using the committee’s anonymised database. We also describe the process of application and approval by the Quality Assurance Committee.

**Results** There were 90 applications, including 10 resubmissions during the 64-month period. Most applicants (77%) were higher trainees; 53% of applicants were from the London deanery; 60% of applications were for research posts and higher degrees (OOPR). Overall, 64% were approved by the committee: 70% for OOPRs and 53% for OOPTs.

**Clinical implications** This paper shows with transparency the breakdown of applications to the Quality Assurance Committee. Around two-thirds of applications to the committee are supported (64%). Relatively few psychiatry trainees (2.5%) have applied for an OOPT or an OOPR over the past 5 years.

The Quality Assurance Committee at the Royal College of Psychiatrists includes a group of consultants across all specialities, a lay member, carer and trainee representative. The committee leads on the review and college approval of trainee’s out of programme training (OOPT) or research (OOPR). Trainees seeking for their OOPT or OOPR to count towards part of their psychiatric training and therefore certificate in clinical training (CCT) apply prospectively to the committee via a structured application form. In this article we will review the process of applying, the outcomes of those that apply, the common pitfalls and problems for those that are unsuccessful and review the demographics of applicants.

## Method

We analysed the numbers of applications, trainee deanery and ethnicity and approval rate of those applying for OOPT or OOPR between January 2008 and April 2013 using the Quality Assurance committee’s anonymised database. We also describe the process of application (see Box 1) and approval by the Quality Assurance Committee.

## Results

### The Applicants

There were 90 applications to the Quality Assurance Committee between January 2008 and April 2013, a 64-month period. This included 10 resubmissions. The descriptive data on core and higher trainees; approval rates; and applications by deanery is described in Table 1.

#### Approval v. non-approval

Of the 90 applications, 58 (64%) were approved and 32 (36%) not approved on the first application. Of the 32 applications not approved, 10 (31%) trainees resubmitted additional evidence or information with 8 (80%) successful in gaining approval. Of the 10 resubmitted, 1 application required minor changes and was approved after personal correspondence between committee members between meetings. Nine (90%) were reviewed at the subsequent committee meeting following resubmission.

#### Core v. higher trainee applicants

Overall most applications were from higher trainees; 77% (70/90). Core trainees included those who were core trainees at the time of applying. These trainees may be applying for OOPT or OOPR in their next post, which may be a higher training post. This may have had an impact on the actual proportion taking OOPT or OOPR in their higher training.

**Box 1** The application process

- Trainee applies for a specific training or research opportunity not part of their programme.
- Trainee starts deanery/LETB process for applying for local approvals; leading to signed LETB form usually by educational supervisor, training programme director and dean or head of school.
- Trainee looks at website: http://www.rcpsych.ac.uk/training/qualityassurance.aspx for timeline of next quality assurance committee meeting.
- If trainee does not want to have OOPT/OOPR to count towards CCT, the trainee need not apply to the Quality Assurance Committee at the College.
- If trainee wants some or all of the OOPT/OOPR to count towards their CCT they then apply to the Quality Assurance Committee using the structured application form on the website in the area on quality assurance of training (http://www.rcpsych.ac.uk/training/qualityassurance.aspx) before starting the new post (i.e. prospectively).
- The trainee includes a covering letter, copy of the signed local approval, the Quality Assurance Committee application form and all the recommended supporting evidence.
- The Committee meet quarterly and review all of the applications. The applications are not anonymised so any members who know the trainee or have a conflict of interest leaves the room.
- The Committee then recommend approval, request for further information or non-approval by letter to the applicant. This includes the number of weeks to months to count towards CCT. Those that are not approved are given the reasons for the decision.
- The approved applications are then forwarded to the General Medical Council for final approval and a letter is sent to the applicant with their amended CCT date.
- Applications that required minor further information are usually reviewed in between meetings online. Applications that require resubmission or are less minor are reviewed at the following quarterly meeting.
- A letter to the applicant, advising the result, is sent by email and post within 1–2 weeks of the quarterly meeting.

**Table 1:** The numbers and success rate of core and higher trainees applying for out of programme training (OOPT) and out of programme research (OOPR) by deanery (local education training board)

Deanery	Total applications, *n*	Applications by core trainee, *n*	Application by higher trainee, *n*	Number supported by college, *n*	Percentage supported by college, %
East Midlands	5	0	5	3	60

East of England	8	2	6	6	75

Kent, Surrey, Sussex	4	0	4	2	50

London	48	12	36	28	58

Mersey	2	0	2	2	100

North Western	4	3	1	3	75

Northern	3	1	2	3	100

Oxford	3	1	2	0	0

Northern Scotland	1	0	1	1	100

Severn	1	0	1	1	100

South-East Scotland	2	0	2	2	100

South-West	1	0	1	1	100

Wales	2	0	2	1	50

Wessex	3	0	3	3	100

West Midlands	1	0	1	1	100

West of Scotland	1	0	1	0	0

Yorkshire and Humber	0	0	0	0	0

Unclear	1	^a^	^a^	1	100

Total	90	19	70	58	64

a. Not able to say from College data.

#### Applications by deanery or local education training board

We analysed the 90 applications across deaneries, now local education training boards (LETBs). The largest deanery in the UK is London in terms of psychiatry trainee numbers. The London deanery applicants made up 53% of applicants (48/90) with the other deaneries making up the remaining. We found that OOPT and OOPR applications were at the same rate in the London deanery (2.5%) as in the overall trainee sample (2.5%); during 2008–2013 the London deanery had 1918 trainees. As a result of the relatively small numbers applying from deaneries outside London, data from all other deaneries was combined for statistical analysis. These data were compared with data from the London deanery. Results from chi-squared analysis (χ^2^(1,*n* = 89) = 0.94, *P* = 0.33) indicates that there was no significant difference in the likelihood of success on an application based on deanery location (London *v.* outside London). This supports the transparency of the application process by deanery.

#### The diversity data

The Quality Assurance Committee reviewed trainee’s ethnicity against application approval for all declared ethnic groups. This was as part of the committee’s process to ensure there was no discrimination as part of the approval process. Declared ethnicity is available for all applications since January 2009. The database includes 76 applications; 95% (*n* = 72) of trainees declared their ethnicity on the OOP application. Table 2 shows that across trainees that declare their ethnicity, 33% (19/57) of White trainees did not have their applications supported *v.* 40% (6/15) of Black and minority ethnic trainees. Data was statistically analysed to see if self-declared ethnic group (White or non-White) was significantly related to the likelihood of success for OOPT and OOPR applications. Chi-squared analysis (χ^2^ (1, *n* = 72) = 0.23, *P* = 0.63) indicates that there was no significant difference in the likelihood of success based on ethnicity. This supports the transparency of the application process by ethnicity.

#### OOPT v. OOPR

The majority of trainee applications 60% (54/90) were for OOPR. The remaining were for OOPT 36% (32/90) and 4% (4/90) were unclear from College databases. A total of 16 of the 54 (30%) OOPR applications were not approved to count towards training or CCT (this included resubmissions). Of the OOPR applications, the majority were for research posts, PhDs and research fellowships. Some diverse applications were approved including a 12-week research post in Ghana and an MSc that counted towards CCT.

Of the 32 OOPT applications to the College, 15 (47%) were unsuccessful in gaining college support towards a CCT. Of the successful 17 applications, 4 (24%) were for forensic training including 3 forensic child and adolescent

**Table 2:** Applications for out of programme training (OOPT) and out of programme research (OOPR) by ethnicity (White and Black and minority ethnic)

Ethnicity Groups	Core trainee, *n*	Higher Trainee, *n*	Applied for OOPT or OOPR, *n*	Supported OOPT or OOPR, *n*	Unsupported OOPT or OOPR, *n*
White (any origin)	907	563	57	38	19

Black and minority ethnic (any origin)	1276	805	15	9	6

Total	2183	1368	72	47	25

psychiatry and a medium secure forensic training post. A total of five (29%) posts were approved for work overseas including South Africa (one), Ghana (two) and Australia (two). Five (29%) fellowship posts were approved in areas as diverse as medical education, quality improvement, healthcare policy and practice fellowships. The remaining posts included working in London at the Maldives High Commission Drugs Policy Unit, clinical lecturer and clinical posts in the UK.

#### Reasons for non-approval

For the 16 OOPR applications not approved, 8 (50%) required further necessary information. For example not including the requested supporting information, not stating how psychotherapy competencies in core training would be met as part of the post or not including previous information on posts. For the remaining rejected applications, reasons for non-approval of core trainees related to issues such as taking OOP too early in core training (contrary to The Gold Guide^1^) (one); taking additional OOPS in core training when already on an academic clinical fellow (ACF) scheme with a 75% clinical post (one). Pitfalls for higher trainees included requesting multiple OOPT or OOPRs to count towards training (three), clinical lecturer posts whose timetables did not meet the clinical training requirements (one), overseas posts that did not meet the curriculum, or trainees who had already completed 24 or more months training at the time of application (two).

For 15 OOPT applications not approved, 6 (40%) were posts in Australia. The main reasons for unsuccessful applications included a lack of evidence particularly around work-based assessments or documenting how assessments would take place. Other reasons for non-approved applications included the following.

- Retrospective and did demonstrate coverage of the curriculum.
- One was for an infant mental health post (children aged 0–3), which is not part of the UK higher training child and adolescent mental health services (CAMHS) curriculum.
- One was for a post at a House of Commons Committee that did not map to the curriculum.
- One was for a post overseas where the duties appeared too junior for equivalence of a higher trainee.
- One post overseas did not demonstrate how the curriculum competencies would be met.
- One had an incorrectly completed application form.
- One was a core trainee before core trainee year 3 (CT3) who had not completed 2 years of core training prior to an OOPT as recommended by the Gold Guide.
- The remaining two required further information to support their application.

#### Trainee psychiatrists v. other colleges trainees

Of the current psychiatry trainees 90 out of 3606 (2.5%) core and higher trainees in psychiatry have applied for OOPT or OOPR. Following request to the other Royal Colleges for comparative data, we have three returns with some information. The responses were from the Royal College of General Practitioners (RCGP), the Royal College of Obstetrics and Gynaecology (RCOG) and The Royal College of Pathologists (RCPath).

The RCGP confirmed that they have no OOPT or OOPR approved to their knowledge. They report that as general practice is a short training programme of 3 years, which includes a mix of training opportunities there is no need to do OOPT. Of those that take time out, for example to work overseas, it is usually recorded as a career break (OOPC) and therefore is dealt with at deanery/LETB level. In terms of OOPR, GP trainees have the opportunity to apply for an academic clinical fellow (ACF) scheme and therefore do not require OOPR during the training period.

The RCOG report that, in 2012, 26 of their trainees had OOPR or OOPT and 62 in 2011. The RCPath report that since 2003, 20 trainees have applied for OOPRs and 7 trainees applied for OOPT and 100% of trainees were approved to count towards training and their CCT. Both colleges did not supply the numbers of trainees in total to compare the rates of OOPT and OOPR with psychiatry

## Discussion

Out of programme training and research remains a fairly uncommon experience with just 2.5% of psychiatry trainees applying for a training or research experience outside of their programme to count towards their CCT. The reasons for this may be that relatively few trainees spend time in out of programme experiences (OOPEs) outside of their programme in psychiatry. However, it may be that, similar to the GP vocational training scheme (VTS) programme, many trainees take time but do not apply for their OOPE to count towards their CCT. Reasons may be that the training programme is relatively short (6–7 years depending on endorsements). It is noted that there are relatively low competition ratios in psychiatry at CT1 and specialty trainee year 4 (ST4) entry over the period analysed. It may be that many trainees do not feel the need to develop their curriculum vitaes in this way through research and OOPT. Qualitative analysis could explore the reasons why in future studies.

A further possible explanation is that trainees do not have an awareness of the process and role of Royal College of Psychiatrists in signposting and supporting applications for OOPT or OOPR. It may be that on a practical level out of programme opportunities and processes within the college and deaneries need to be more actively promoted.

The analysis shows that about two-thirds of trainees (64%) who apply for OOPT or OOPR are successful in getting approval by the Quality Assurance Committee. The Committee noted that a proportion of approvals were incomplete and required further information or evidence of mapping to the curriculum or training programme. We have simplified the application system by creating a form that prompts trainees to complete all of the required information. This went live in October 2012 on the College website. The updated guidance document is available on the College website.

There are relatively low numbers of OOPR applications (54 over 64 months). This suggests that there is a relatively low interest in research opportunities outside of the academic clinical fellow scheme; just 1.5% (54/3606) trainees have had the opportunity to be actively involved in a full-time research opportunity as OOPR. This suggests that relatively few trainees will have exposure to practical research experience, which may be of some concern to academics and the profession. Medicine and allied health sciences is based on the principles of evidence-based practice, where current best evidence is utilised in making decisions about the care of individual patients, therefore research is critical to developing psychiatry’s evidence base and practice.^2^

It is noted there are relatively high numbers of applications from the London deanery (53%), compared with all of the other deaneries. The data demonstrate that London, compared with the UK as a whole, has the same rate of uptake of OOPT and OOPR, when taking into account the total number of psychiatry trainees in the deaneries. Despite a large number of applications from London, applications were not significantly more or less likely to be approved. This supports the transparency of the application and review process. This was also found for ethnicity supporting issues around equal opportunities and diversity. The larger number (not rate) of OOPTs and OOPRs in London suggests that there may be a culture there that encourages trainees to develop their expertise through OOPTs or OOPRs, which is not the case in other regions to the same extent. However, it may be because a large proportion of the research and training opportunities available out of programme are in London as a result of the high number of expert centres and universities. It may be that there is a supportive ‘hidden curriculum’ in London; led by supervisors with explicit links to these centres in signposting, promoting and supporting trainees in research and additional training.

### Implications

Overall, although only a small proportion of trainees apply for OOPT or OOPR. Our analysis indicates that the process of its quality assurance is transparent in terms of applicants by ethnicity or deanery. The majority of applications overall are successful. Future research should examine how psychiatry compares with other specialisms in more detail and the reasons why low percentages of psychiatry trainees are applying for OOPT or OOPR. However, there is a lack of easily accessible comparative data making generalisations across specialisms difficult.
